# When science meets entrepreneurship

**DOI:** 10.12688/openreseurope.15318.1

**Published:** 2023-01-13

**Authors:** Dara Satrio, Vanessa Rijkeboer, Angelica Reitsma, Edo Roos Lindgreen, Katja C. Wolthers, Dasja Pajkrt

**Affiliations:** 1 Academic Medical Center of the University of Amsterdam, Amsterdam, The Netherlands; 2 Department of Economics and Business Administration, University of Amsterdam, Amsterdam, The Netherlands

**Keywords:** Early Stage Researchers, Scientists, pre-MBA program, Innovative Training Network, Researchers.

## Abstract

Science industries, such as the health and medical industry, are experiencing increases in competition regarding commercializing, patenting, and funding of scientific outputs. As such, scientists are facing increased expectation to engage in academic entrepreneurship. OrganoVIR (Organoids for Virus Research) is a Horizon2020 Innovative Training Network (ITN) that aims to train Early Stage Researchers (ESRs) to lead innovation in the field of human organoids for virus research. To assist them in this process, OrganoVIR introduced a pre-Master of Business Administration program that introduced OrganoVIR’s ESRs to the tools and knowledge necessary to navigate the competitive industry. In this article, we describe this innovative pre-Master of Business Administration program and highlight the importance as well as the need for having a pre-Master of Business Administration programs in a scientific training network.

## Disclaimer

The views expressed in this article are those of the author(s). Publication in Open Research Europe does not imply endorsement of the European Commission.

## Plain language summary

The science industry is arguably one of the most competitive industries in the world. Nowadays, scientists are competing to commercialize, patent their research and obtain funding for future research. OrganoVIR, a Horizon2020 Innovative Training Network (ITN) under the EU Horizon 2020 research program, trains 15 Early Stage researchers (ESRs) to lead innovation in the field of human organoids for virology in Europe. Through one of its training programs, the innovative OrganoVIR pre-Master of Business Administration program aims to introduce the next generation of scientists to the European labour market. The implementation of this program marked the first time that a pre-Master of Business Administration (pre-MBA) program was successfully incorporated in a scientific training network. The OrganoVIR pre-MBA program is curated through collaboration with the Amsterdam Business School in the Netherlands. The program consisted of five modules: Process Improvement in Healthcare, Competitive Strategy, Marketing Strategy, Crisis Management, Navigating Life in Pharma Organizations and Writing a Business Plan. With this article, we aim to demonstrate the importance of integrating a pre-MBA program into a scientific ITN. Furthermore, we would like to encourage other ITNs operating within the science industry to also integrate a pre-MBA program into their scientific training program.

## Introduction

OrganoVIR (Organoids for Virus Research), coordinated by Dr. Katja Wolthers and Prof. Dr. Dasja Pajkrt, is a Horizon2020 Innovative Training Network (ITN) under the EU Horizon 2020 research program. OrganoVIR aims to make human organoids a superior model for virus research and to transform the virology landscape.

OrganoVIR trains 15 Early Stage Researchers (ESRs) to lead innovation in the field of human organoids for virology in Europe. The OrganoVIR training program consists of three pillars that build up scientific, human and business skills for applying organoids for virus research. The three training pillars will enable the ESRs to lead innovation in the field of organoids for virus research and to hold a unique position within the European labour market.

Within OrganoVIR, scientific academic skills are being translated through peer-reviewed scientific publications. Furthermore, the training on human skills is provided by a unique personal development plan (PDP) which was previously reported in *Humanizing Science: seven actions for PhD students to become next generation, future-proof scientist* ( Valks *et al*., 2022). Moreover, the business competences are provided by the OrganoVIR pre-Master of Business Administration (pre-MBA) programme. The pre-MBA aims to train ESRs in entrepreneurial and managerial skills, as most young researchers end up working in a commercial setting. 

Today, scientists are becoming more proactive in commercializing their research results through patenting, licensing, consulting, and firm founding ( Krabel & Mueller, 2009). Since the Bayh-Doyle Act ^1^ was passed in 1980 in the United States of America (U.S.), there has been an apparent increase in the commercialization of science ( Siegel & Wright, 2015). In the following years, there was a noticeable surge in university licensing, patenting and start-up creation in the USA which could also be seen in Europe and Asia, Australia, and Canada ( Grimaldi *et al*., 2011). Due to the competition, academic scientists, including faculty, postdoctoral fellows (postdocs), and graduate students are facing increasing expectations to engage in academic entrepreneurship ( Siegel & Wright, 2015).

Currently, no scientific training network has implemented a pre-MBA as part of its training program. However, upon designing the OrganoVIR project, Wolthers and Pajkrt recognized that a pre-MBA program is needed to equip OrganoVIR’s ESRs with knowledge of entrepreneurship and business analytics. The OrganoVIR pre-MBA program, a training program that was curated through collaboration with the Amsterdam Business School in the Netherlands, was implemented into the OrganoVIR training program to deliver next-generation scientists to the competitive labour market.

The OrganoVIR pre-MBA program has a different training purpose compared to the OrganoVIR PDP. In a previous study about the OrganoVIR PDP ( Valks *et al*., 2022), we highlighted how the latter focuses on training the *human skills* of the ESRs by focusing on topics such as: 1) well-being; 2) purpose in life; 3) identity; 4) values and beliefs; 5) emotional competences; 6) leadership behaviour; and 7) ethical dilemmas. The OrganoVIR pre-MBA programme focuses on the education of OrganoVIR’s ESRs with the aim to equip them with entrepreneurial and business analytic skills. This way, this next generation of scientists will be able to operate within commercial settings. The pre-MBA program consists of the following modules: 1) Process Improvement in Healthcare, 2) Competitive Strategy, 3) Marketing Strategy, 4) Crisis Management and Navigating Life in Pharma Organizations and 5) Writing a Business Plan.

In this article, we aim to describe the various modules of the OrganoVIR pre-MBA program and highlight the importance as well as the need for having a pre-MBA program in a scientific training network.

### Process improvement in healthcare

The OrganoVIR pre-MBA program began with the first module, Process Improvement in Healthcare. Managing a business involves identifying, analyzing and developing improvement opportunities in processes and the organization. To stand out in the European labour market, our ESRs will need to develop professional skills in problem analysis, problem-solving and decision-making.

Within this module, OrganoVIR’s ESRs were trained in the following skills; increasing the ability to manage business improvement in the form of a project, dealing with the political force field in which such projects take place, rational problem solving and decision analysis, using quantitative analysis and decision techniques, applying the principles of the Lean Six Sigma.

The Lean Six Sigma, a structure for organizing improvement activities, incorporates the most effective approaches which offer a structure to establish an effective and continuous improvement (De Mast *et al*., 2015). In this module, OrganoVIR’s ESRs learn to solve problems by using their professional, science-like skills. OrganoVIR’s ESRs were guided to analyse the causes of problems, understand quantitative problem definitions and perform data-based diagnoses.

The methodological principles underlying Lean Six Sigma were integrated with a collection of tools and techniques into one roadmap, called DMAIC (Define, Measure, Analyse, Improve, and Control). Each lecture focuses on the different tools and techniques of Lean Six Sigma. Additionally, topics regarding the organization of process improvement were discussed including; organizational structure, project management, deployment, political force field, and managing resistance.

### Competitive strategy

The science industry is competitive regarding influence, faculty positions, funding, publications, and students ( Bok, 2003). Over the years, numerous processes, theories and tools have been developed to help organizations compete successfully. This results in two outcomes; 1) a traditional strategic approach that relies on analysis and planning and 2) an alternative approach that relies on creativity, learning, co-creation, and design.

This module revolved around two questions. First, “why do some organizations have advantages over their competitors?” and second, “What can managers do to build and maintain such competitive advantages?”. To answer these questions, the module introduced topics such as planned and emergent strategy, strategy process (activating, mapping, assessing, innovating and formulating), and the difference between inside-out and outside-in approaches to strategy.

Through interactive sessions in this module, OrganoVIR’s ESRs answered the two questions by learning to apply strategic processes, theories and tools to their own case study. In essence, during this module the ESRs learned the following: 1) how strategy, competition, value creation and value capturing drive the performance of organizations; 2) the main processes, theories and tools of competitive strategy; 3) the main strengths and weaknesses of these processes, theories and tools; 4) how to diagnose strategy issues in case-based and real-life organizations; 5) utilise the processes, theories and tools in order to improve the strategy of case-based and real-life organizations.

### Marketing strategy

The healthcare industry is constantly transitioning. In this phase of transition in the industry, the role of marketing strategy is changing as well. Although traditional marketing focused on identifying the market, customer segment, and products, it is also important to make a difference and to attract customers and patients with an appealing value proposition. Currently, marketing involves understanding customer behaviour and their emotional needs, providing a compelling service that is equivalent to valuable experience and designing a go-to-market strategy.

In the Marketing Strategy module, OrganoVIR’s ESRs learned to: 1) recall and recognize the marketing frameworks, ideas and principles; 2) comprehend and translate the marketing and behaviour frameworks to a business and healthcare challenge; 3) select and synthesize the best marketing strategy tools and tactics to tackle a patient, product, service; 4) evaluate and translate perspectives from other industries to healthcare; 5) apply knowledge of marketing strategy, innovation and behaviour.

This module provided the knowledge and the skills to analyze, design and execute a marketing strategy. Within the course, several practical marketing strategy topics were addressed including: 1) patient-driven marketing; 2) design value proposition and (digital) healthcare services on the patient journey; 3) innovation in care – digital care services and innovation; 4) purpose-led marketing; 5) building brands in healthcare, brand experience and equity.

### Crisis management

Every healthcare industry encounters a crisis at some point. During a crisis, organizations temporarily go “out of control”. Crisis management offers an alternative and makes it possible for an organization to maintain control during a crisis.

The first masterclass in the Crisis Management module focused on crises and the core principles of crisis management. In this masterclass, the ESRs became acquainted with various crises they might face and how to prepare for a crisis. Questions such as “how to diagnose a crisis?”, “what are the main challenges in crisis management?”, “how does a crisis impact an organization” and “how to prepare for a crisis?” were addressed in this masterclass.

In the second masterclass of this module, the ESRs learned how to manage a crisis. Focusing on creating ownership and leadership, the ESRs were introduced to the core aspects of the Crisis Management Model and the key characteristics of great crisis leaders. Furthermore, the ESRs became acquainted with an effective crisis meeting approach and experienced the dynamics of a crisis during a brief crisis simulation. This masterclass addressed questions such as “how do you manage a crisis?”, “which leadership skills are needed during a crisis?” and “how to make quick and well-considered decisions?”.

### Navigating life in pharma organizations

The pharmaceutical industry is a rapidly growing landscape. In the U.S. alone, pharmaceutical industries develop a variety of expensive new drugs every year which contribute to an increase in healthcare costs for the private sector and the federal government ( Congressional Budget Office, 2021). The increase in scientific breakthroughs may create opportunities for pharma organizations, the intensifying competition also means that these organizations must focus on meeting the needs of patients and create a sense of personalization to keep their products in the market ( Aronowitz *et al*., 2022).

To help our ESRs navigate their lives through the commercial pharmaceutical industry, we began this module with the first masterclass which focused on the research and development (R&D) aspect of pharmaceutical organizations. This masterclass explored the history of the pharmaceutical industry and the pharmaceutical innovation process. During this masterclass, the ESRs learned to diagnose R&D inefficiency and potential remedies, rethink the innovation process and have discussions about existing innovation processes in healthcare.

The second masterclass focused on the landscape of the pharmaceutical industry, inter-organizational collaboration and entrepreneurship. OrganoVIR’s ESRs learned the importance of collaboration between in-house and external R&D, the role of small and medium-sized organizations in the biotech industry, types of collaborations in the pharma industry, and funding opportunities for entrepreneurs. Topics such as the Stage-Gate process ^2^, its implementation in the development of medical devices, and innovation in healthcare were discussed.

The third masterclass focused on social value creation, ethics, and sustainability. The masterclass began with an introduction to ethical theories and ethical dilemmas in healthcare. Ethics does not refer to ethical guidelines, but more to the philosophical questions about ethics in healthcare practices (for example, who to treat first?). Furthermore, OrganoVIR’s ESRs learned how the Sustainable Development Goals (SDGs) affect the healthcare landscape, the difference between social value and financial value in healthcare, and the role of patients in the healthcare innovation process.

### Writing a business plan

To conclude the OrganoVIR pre-MBA program, the last module focused on writing a business plan. Although writing a successful business plan can be stimulating, it can also be challenging and requires knowledge of several entrepreneurship concepts and steps. The final module introduced the basic principles of entrepreneurship and the main tools that can be used to set up a new company and write a business plan.

Upon completion of this module, we found that our ESRs were able to identify and delineate the fundamental steps in the creation of a new company, from idea generation to financing. Furthermore, our ESRs learned to develop an effective business model and business plan for the new development of a new company. Finally, we also found that our ESRs were able to collaborate in a team when developing their business.

## Conclusions and recommendations

The scientific industry is competitive. In this article, we have addressed how the global competition for university licensing, patenting and creation of start-up companies have increased since the Patent and Trademark act was introduced in 1980. OrganoVIR understands that implementing the pre-MBA program into a scientific training program will guide the next generation of researchers to become leading entrepreneurs in the science industry. Through the pre-MBA program, OrganoVIR’s ESRs were trained in the skills involved in managing a business such as developing competitive and marketing strategies, navigating through crisis and organizations and writing a business plan. We believe that in order to excel in the competitive labour market, the next generation of scientists must not only be trained in science but also in human skills and entrepreneurial skills. Hence, with this article, we encourage other scientific training networks to also implement a pre-MBA program as part of their training program.

## Data Availability

No data are associated with this article.

## Footnotes

^1^ The Bayh-Dole Act, formerly known as the Patent and Trademark Act Amendments, is a federal law that was introduced in 1980 that enables universities, non-profit research institutions and small businesses to own, patent and commercialize inventions developed under federally funded research programs within their organizations.

^2^ *State-Gate Process* is a conceptual and operational map for moving new product projects from “idea” stage to launch. It is the blueprint for managing the new product development (NPD) process.
