# Supplementary material for: When science meets entrepreneurship
Source: Open Res Eur. 2023 Jan 13;3:9. [Version 1] doi: 10.12688/openreseurope.15318.1 (PMC10521095; doi:10.12688/openreseurope.15318.1)
Supplement: Peer review history

# Reviewer report: Reviewer response for version 1

**DOI:** 10.21956/openreseurope.16560.r34068

**Published:** 2023-09-18

**Authors:** Sila Kaya-Capocci

**Affiliations:** 1 Agri Ibrahim Cecen University, Ağrı, Eastern Anatolia, Turkey

The research aimed to aims to train Early Stage Researchers (ESRs) as part of a Horizon 2020 project to lead innovation in the field of human organoids for virus research. The research sounds interesting but needs clarifications and literature support. Some parts do not flow well due to the unclear links and the jumps between general and specific information. A funnel model should be adapted moving from general to specific information. A clear structure is recommended in the pdf attached.

 The developed pre-MBA programme includes five modules. However, rationalisation of the modules based on the current research is not enough. In each module, the need and the importance should be highlighted more in the light of the current research. In each module, the strategies used to reach the learning outcomes are not clearly stated. Currently, if I want to develop and implement a module based on this research, I am not sure how I should structure the module, what skills I should focus on and what strategies I should use, how long the modules should be or how many masterclasses the module should include. Therefore, I do not have a clear guideline. Furthermore:

- Three types of skill sets are mentioned in the introduction (scientific academic, human, and business). Can you please introduce what skills are focused in each module?
- I recommend adding a sentence at the end of each section introducing the activities applied in each module. Currently, the aims of the modules are clear but how it is targeted to be achieved (methods/strategies/approaches) is vague. These would really benefit the audience.
- To increase the validity and reliability, how the researchers know the learning was improved should be clarified, and the details mentioned above should be provided.

Further details about the comments and recommendations can be found on the pdf document attached here.

 When the parts are clarified a bit more and enriched with recent references, the research is worth reading. I believe the research can contribute to the field.

Where applicable, are recommendations and next steps explained clearly for others to follow? (Please consider whether others in the research community would be able to implement guidelines or recommendations and/or constructively engage in the debate)

No

Does the article adequately reference differing views and opinions?

Partly

Are all factual statements correct, and are statements and arguments made adequately supported by citations?

Partly

Is the rationale for the Open Letter provided in sufficient detail? (Please consider whether existing challenges in the field are outlined clearly and whether the purpose of the letter is explained)

Yes

Is the Open Letter written in accessible language? (Please consider whether all subject-specific terms, concepts and abbreviations are explained)

Yes

Reviewer Expertise:

My area of expertise includes STEM and enterprise education.

I confirm that I have read this submission and believe that I have an appropriate level of expertise to confirm that it is of an acceptable scientific standard, however I have significant reservations, as outlined above.

# Reviewer report: Reviewer response for version 1

**DOI:** 10.21956/openreseurope.16560.r34610

**Published:** 2023-09-18

**Authors:** Noorlizawati Abd Rahim

**Affiliations:** 1 Razak Faculty of Technology and Informatics, Universiti Teknologi Malaysia, Kuala Lumpur, Kuala Lumpur, Malaysia

**Summary:**

 The article discusses the OrganoVIR (Organoids for Virus Research) program, a Horizon2020 Innovative Training Network (ITN) that aims to train Early Stage Researchers (ESRs) in the field of human organoids for virus research. The focus is on the introduction of a pre-Master of Business Administration (pre-MBA) program within OrganoVIR to equip ESRs with entrepreneurial and managerial skills, acknowledging the increasing expectation for scientists to engage in academic entrepreneurship. The program is unique in its approach, as no other scientific training network has implemented a pre-MBA as part of its training. The pre-MBA program consists of five modules: Process Improvement in Healthcare, Competitive Strategy, Marketing Strategy, Crisis Management, and Navigating Life in Pharma Organizations. Additionally, a module on Writing a Business Plan concludes the program. Each module aims to provide ESRs with practical skills and knowledge needed to excel in commercial settings.

**Recommendations**

1. While the article provides a comprehensive overview of the pre-MBA program, it could benefit from including concrete outcomes or success stories of ESRs who have completed the program. Providing data on how the program has benefited participants and their subsequent careers would strengthen the article's argument for the importance of pre-MBA programs.
2. To further emphasize the uniqueness and effectiveness of the OrganoVIR pre-MBA program, the authors could compare it to alternative approaches or models for training scientists in entrepreneurship. This could include discussing the strengths and weaknesses of different approaches and why the pre-MBA model was chosen.
3. The article acknowledges the need for pre-MBA programs but does not directly address potential criticisms or concerns. For a more balanced perspective, the authors could discuss any drawbacks or challenges associated with integrating business education into scientific training and propose strategies to mitigate these issues.

Where applicable, are recommendations and next steps explained clearly for others to follow? (Please consider whether others in the research community would be able to implement guidelines or recommendations and/or constructively engage in the debate)

Partly

Does the article adequately reference differing views and opinions?

Partly

Are all factual statements correct, and are statements and arguments made adequately supported by citations?

Yes

Is the rationale for the Open Letter provided in sufficient detail? (Please consider whether existing challenges in the field are outlined clearly and whether the purpose of the letter is explained)

Yes

Is the Open Letter written in accessible language? (Please consider whether all subject-specific terms, concepts and abbreviations are explained)

Yes

Reviewer Expertise:

academic/technology entrepreneurship, entrepreneurial leadership, entrepreneurship education

I confirm that I have read this submission and believe that I have an appropriate level of expertise to confirm that it is of an acceptable scientific standard.

# Reviewer report: Reviewer response for version 1

**DOI:** 10.21956/openreseurope.16560.r34609

**Published:** 2023-09-08

**Authors:** Danny Soetanto

**Affiliations:** 1 Lancaster University Management School, Lancaster, UK

I appreciate the privilege of reviewing the submission titled 'When Science Meets Entrepreneurship' and would like to extend my gratitude for this opportunity. This paper delves into the compelling intersection of science and entrepreneurship, with a specific focus on the induction of an entrepreneurship curriculum for Early-Stage Researchers (ESRs). While the paper contains valuable insights, I believe there is potential for further enhancement to truly elevate its impact and contribution to the field. In this feedback, I aim to provide constructive suggestions and insights to help refine and strengthen the paper.

 First, I believe the paper could benefit from greater clarity regarding its research questions and the contributions it aims to offer. Clarifying how this paper fills gaps in existing knowledge would enhance its overall contribution. It would be nice if there is a clear explanation how the current programme addresses the challenges faced by academic scientists. Alternatively, if the authors could devote more attention to identifying current debates in entrepreneurship education for scientists and explaining how the newly designed pre-MBA modules can address these challenges, the paper would undoubtedly provide greater value.

 Another avenue to strengthen the paper's contribution is by introducing novelty. While I agree with the paper's assertion that introducing entrepreneurship to pre-MBA courses is relatively new, it is worth noting that entrepreneurship is already a common skill taught in many MBA programs and training courses for academic entrepreneurship. Given the shortage of entrepreneurship skills and knowledge among scientists, coupled with a growing emphasis on knowledge commercialization in Europe, numerous initiatives have emerged to enrich and expand the entrepreneurship capabilities of researchers, academics, and scientists. I believe it is crucial to distinguish this program from previous or existing initiatives. Also it would be advantageous to highlight the uniqueness of the proposed program within this broader context, such as its relevance to the Organovir project.

 Once the research objectives are well-established, the next step is to find a theoretical framework that can serve as the foundation for the curriculum design. This does not necessarily mean the authors must find pedagogical theories to support the design, but they can explain the rationale behind selecting particular modules contents. Since the pre-MBA program includes modules such as "Process Improvement in Healthcare," "Marketing," and "Navigating Life," it would be highly informative to understand the rationale behind the development of these modules and how they integrate within the entrepreneurial narrative of the program. Moreover, the section on "Writing a Business Plan" deserves more in-depth explanation. Entrepreneurship pedagogy has evolved significantly over the past few decades, with various contemporary approaches that go beyond mere business plan writing. I am confident that this module incorporates unique approaches or contextual elements that are worth showcasing.

 Regarding the conclusion, it would be valuable to include an evaluation of the program and provide recommendations. As far as I can discern, there have been no significant breakthroughs in teaching entrepreneurship to scientists in general. Therefore, if this pre-MBA program has specific measurable criteria or successful stories, it would be worthwhile to mention them in this section.

 Finally, the introduction appears somewhat lengthy compared to the overall content. A more concise introduction that clearly communicates the research objectives, reasons for conducting the research, and the intended contributions of the paper would be preferable.

Where applicable, are recommendations and next steps explained clearly for others to follow? (Please consider whether others in the research community would be able to implement guidelines or recommendations and/or constructively engage in the debate)

Partly

Does the article adequately reference differing views and opinions?

Partly

Are all factual statements correct, and are statements and arguments made adequately supported by citations?

Yes

Is the rationale for the Open Letter provided in sufficient detail? (Please consider whether existing challenges in the field are outlined clearly and whether the purpose of the letter is explained)

Partly

Is the Open Letter written in accessible language? (Please consider whether all subject-specific terms, concepts and abbreviations are explained)

Yes

Reviewer Expertise:

Entrepreneurship, Academic entrepreneurship.

I confirm that I have read this submission and believe that I have an appropriate level of expertise to confirm that it is of an acceptable scientific standard, however I have significant reservations, as outlined above.

# Reviewer report: Reviewer response for version 1

**DOI:** 10.21956/openreseurope.16560.r34074

**Published:** 2023-08-22

**Authors:** James Dzisah

**Affiliations:** 1 University of Ghana, Accra, Greater Accra Region, Ghana

This brief article describes the ongoing attempts by various policymakers to bridge the gap between knowledge discovery and application. It follows the Edinburgh-Standford link which was directed at harnessing the entrepreneurial and innovation ecosystem of the Silicon Valley experts through collaborative research linkages with their Scottish counterparts. 

 In broad strokes, the paper describes a variant of the quest to embed business skills in research practices of early career scientists. The novelty here is the introduction of pre-MBA program to anchor the scientific training networks.  

 Though very promising, as the pre-MBA program practicalizes 'science-business', it remains to be seen how the transition from pre-MBA to actual practice works for the early scientists. In spite of this reservation, this paper provides a translatable approach that is likely to become a cost effective model, compared to the integration of a fully-fledged MBA masterclass model, to moving knowledge to the marketplace.

Where applicable, are recommendations and next steps explained clearly for others to follow? (Please consider whether others in the research community would be able to implement guidelines or recommendations and/or constructively engage in the debate)

Yes

Does the article adequately reference differing views and opinions?

Yes

Are all factual statements correct, and are statements and arguments made adequately supported by citations?

Yes

Is the rationale for the Open Letter provided in sufficient detail? (Please consider whether existing challenges in the field are outlined clearly and whether the purpose of the letter is explained)

Yes

Is the Open Letter written in accessible language? (Please consider whether all subject-specific terms, concepts and abbreviations are explained)

Yes

Reviewer Expertise:

Triple Helix, Globalisation and Innovation Policy, Public Policy, Sociology of Science and Knowledge

I confirm that I have read this submission and believe that I have an appropriate level of expertise to confirm that it is of an acceptable scientific standard.
